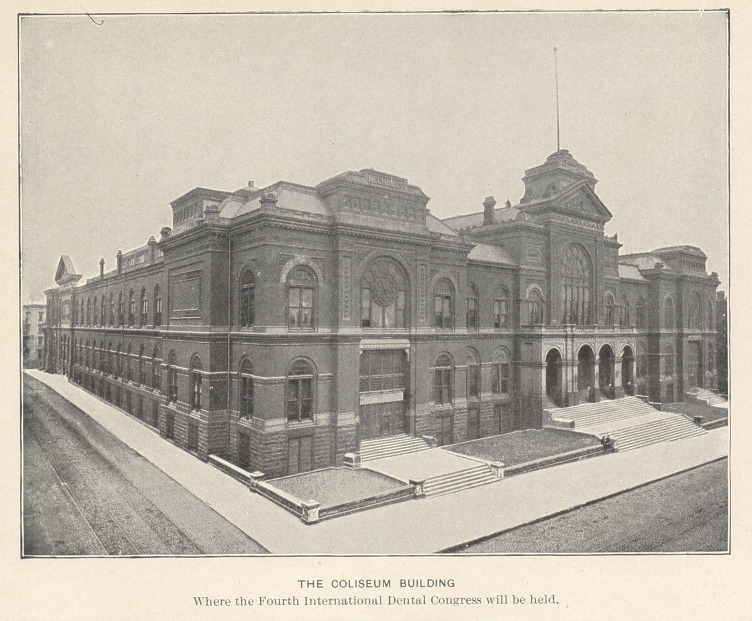# Current News

**Published:** 1904-06

**Authors:** 


*Committee.*

### Current News.

### PENNSYLVANIA STATE DENTAL SOCIETY.

The Pennsylvania State Dental Society will hold its thirty-sixth annual meeting at Wilkes-Barre, Pa., on the 12th, 13th, and 14th of July, 1904.

George W. Cupit,
*Secretary.*

### UNIVERSAL EXPOSITION.

DAVID R. FRANCIS, *President.*

HOWARD J. ROGERS, *Director of Congresses.*

### FOURTH INTERNATIONAL DENTAL CONGRESS.

(*St. Louis, August 29 to September 3, 1904.*)

*Committee of Organization.*
H. J. BURKHART, *Chairman.*
E. C. KIRK, *Secretary,*
Lock Box 1615, Philadelphia.

*Local Committee of Arrangement.*

WILLIAM CONRAD, *Chairman.*

SAM. T. BASSETT, *Secretary,*

457 Century Bldg., St. Louis.

WE, the Committee on Local Information, submit the following facts, important for the success of the Fourth International Dental Congress:

Every member of the profession who has at heart the welfare of dentistry should give it support financially and by action.

We, as American members of the dental profession, would well keep in mind that the Congress will be held in our great country, and pride should prompt us to action to make the meeting one of great magnitude and far excel its predecessors.

Let every dentist take hold with a will and push it to a successful issue.

This Congress should be of great interest to all of the profession. There is planned a monster clinic from the best clinicians of the world, scientific papers from the pens of the most noted and scholarly men of the profession, and an exhibit to excel all others.

The section meetings, the clinics, and the exhibits will be held in the great Coliseum, where ample accommodations are secured.

Hotel Jefferson, the general head-quarters of the Dental Congress, one of the most fashionable and complete hotels in the West, if not in America, is located on Twelfth Street, one block from the Coliseum. Hotels of St. Louis will be sufficient to meet all requirements.

The Information Bureau of the Exposition has a list of ninety-seven well-established hotels now doing business in St. Louis, with a capacity for twenty-one thousand guests, at prices ranging from fifty cents a day up on the European plan, and from one dollar a day up on the American plan. These established hotels have been supplemented during the year 1903 by thirty-five new permanent hotels now opening or about to open, increasing the permanent hotel capacity to forty-seven thousand guests, at prices ranging from one dollar a day up. The Exposition management holds the signed agreement of the leading hotels that " rates shall not be increased during the World's Fair period." Prices are now lower in St. Louis than in any other city for similar hotel accommodation and service.

The Exposition Information Bureau list of one hundred and thirty-two permanent hotels includes only the better class. There are now one hundred and seventy-three hotels, large and small, in operation in the city, and the new hotel enterprises being inaugurated justify the belief that the number will reach two hundred and fifty before the opening day of the World's Fair.

Besides hotels with accommodations for more than two hundred thousand guests, the Exposition Information Bureau has a list of boarding-houses and rooming-houses of respectable character on the street-car lines with lodgings for fifteen thousand guests, and a list of private houses that will let rooms for twenty thousand persons.

All over the city, apartment-houses and rooming-houses are available for those who prefer rooms away from crowds, with meals at the restaurants.

There are four hundred and eighty-five restaurants in St. Louis that have a reputation for good fare, good service, cleanliness, and moderate prices. Twenty of these four hundred and eighty-five restaurants can take care of forty thousand patrons.

The climate of St. Louis is temperate in summer and most delightful in the spring and fall. It is the most central and accessible of the four large cities of the United States, twenty-seven more railways entering it, and passenger steamers on the Mississippi reaching it from north and south.

World's Fair cheap rates on railways and steamboats will be offered during the whole Exposition season. The New England Passenger Association, Trunk Line Association, Central Passenger Association, and Southeastern Passenger Association have adopted the following rates, which will be on sale from April 15:

Season tickets for eighty per cent. of double one fare, good to return until December 15.

For sixty days, one and one-third fare, not good to return after December 15.

For ten days, one fare plus two dollars, from points within two hundred and fifty miles of St. Louis.

For fifteen days, one fare plus two dollars, from points over two hundred and fifty miles from St. Louis.

St. Louis is the fourth city of the United States in point of population, having seven hundred and fifty thousand people. Certainly, no city is more attractive with interest for the student of nature, science, history, etc. There are twenty-four public parks containing over two thousand one hundred acres, all well improved.

The World's Fair Grounds lie five miles from the river on the western edge of the city, and are reached quickly and comfortably by steam railway and fast trolley lines.

The visitors reach the city through the largest and most beautiful railway station in the world.

Thirty-two tracks run into the station side by side, and the midway or glass-roofed hall in front of the gates to the trains will hold thirty thousand people.

Most of the hotels, except the temporary ones near the World's Fair Grounds, are within ten minutes' ride of the station in the heart of the business district. Street cars, reaching all of the hotels for one fare, run by the station, and the cab, carriage, and baggage system is excellent.

The great Music Hall and Coliseum Building that is secured for the meetings of the International Dental Congress is down town, on Olive Street, between Thirteenth and Fourteenth Streets, within easy walking distance of Union Station, the hotels, and the business district. It has a seating capacity for eight thousand people, with section rooms that will be arranged for the various committees and exhibitors.

Naturally, every member attending the Fourth International Dental Congress will wish to see the World's Fair. This will be the most magnificent the world has ever seen, and it will probably be the last great exposition to be held in this country for many years, because of the enormous expenditure of labor and money attending it.

Congressman Bartholdt, in a recent speech made before Congress, said, "The Universal Exposition at St. Louis is the apotheosis of centuries of civilization. It is the culminating perfection of those wonderful international spectacles which have served to impress on our minds that it is good to be a living participant in the glories of this world.

"A decade of human achievement has elapsed since the Columbian pageantry of progress at Chicago. Every American who saw the 'White City' thrilled with the thought that the nations of the earth had assembled in the greatest republic to do homage to the genius of enlightenment.

"Triumphs of the emperors of imperial Rome were but the mock pomp of childish fancies compared to the triumphs of peace as celebrated by such a labor of love at St. Louis. On the May day of this year the gates of welcome will be flung wide open, and the vision of the century will then unfold its prophetic beauty for the uplifting of humanity.

"All in all, the Universal Exposition of 1904 will be the sensational climax of the twentieth century, the grandest victory of peace and civilization, the greatest triumph human genius has yet achieved. To millions of its visitors it will be an academy of learning, an inspiration, and an inexhaustible source of genuine delight, and the memories of the Ivory City will live and bear fruit in ages yet to come."

We appeal to all reputable, legally qualified practitioners of dentistry to interest themselves in the success of this great international meeting.

*Let every dentist in America put forth his greatest effort to advance the cause. Make application at once through your State chairman for a membership certificate. See that your fellow-practitioner is enrolled, and then, and not till then, will you have done your full duty.*

The membership fee to the Congress will be ten dollars. It will entitle the holder to the official badge and all the rights and privileges of the Congress. He will also receive one copy of the transactions. Without a membership certificate, it will be impossible to get into the general meeting, sections, or clinics, or attend any of the various entertainments given during the Congress.

This great Congress presents to the American dentist an opportunity to see and hear the brightest and most learned men of the profession from all parts of the world,—men of international reputation that shine as clinicians and men of great renown of the inventive turn of mind. This international meeting will be the Mecca of the profession of the world. We will all receive new light and be stimulated to a higher appreciation of our noble profession.

The Local Committee of Arrangements and Reception, with its various minor committees, are working with increasing energy, and will be ready to meet you and extend you a most hearty welcome.

Any further information can be obtained from the committee.

D. O. M. LeCron, *Chairman,* Missouri Trust Building,
Max Fendler, *Secretary,* Missouri Trust Building,
George H. Gibson,
H. F. D'Oench,
G. L. Kitchen,
S. H. Voyles,
Orem H. Manhard,
*Permanent Local Committee and Bureau of Information.*

---

### NATIONAL ASSOCIATION OF DENTAL EXAMINERS.

The National Association of Dental Examiners will hold their annual meeting in the Coliseum Building, corner of Thirteenth and Olive Streets, St. Louis, Mo., on the 25th, 26th, and 27th of August, beginning promptly at 10 A.M. Telephone and telegraph offices in the building. Hotel accommodations will be secured for the members. Special railroad rates will be secured for those in the East desiring to attend, trains leaving on the 23d from New York.

The Committee on Railroad Accommodations for the East have made arrangements for fast through Pullman service to St. Louis from New York with the Delaware and Lackawanna Railroad. Two special Pullman cars will leave New York Tuesday, August 23, at 10 A.M. The cost of our excursion including berth each way will be $35.50. A proportionate reduction is made for those going from Buffalo, Toledo, Fort Wayne, and cities on the line connecting with the Wabash Railroad. To those desiring to go in the special cars, send notice to Charles A. Meeker, D.D.S., Secretary of the National Association Dental Examiners, or to Guy Adams, General Passenger Agent of the Delaware and Lackawana Railroad. Accommodations have been secured for the National Association of Dental Examiners at the Franklin Hotel, northwest corner of Sarah and Westminster Place, with rates from $1.50 to $6.00 per day, European plan. Hotel first-class. Secure rooms by writing to E. C. Dunnavant, St. Louis Service Company, Seventh and Olive Streets, St. Louis, Mo.

Charles A. Meeker, D.D.S.,
*Secretary.*

---

### FOURTH INTERNATIONAL DENTAL CONGRESS, ST. LOUIS, MO., AUGUST 29 TO SEPTEMBER 3, 1904.

*Committee of Organization.*—H. J. Burkhart, Chairman, Batavia, N. Y.; E. C. Kirk, Secretary, Lock Box 1615, Philadelphia, Pa.; R. H. Hofheinz, Wm. Carr, W. E. Boardman, V. E. Turner, J. Y. Crawford, M. F. Finley, J. W. David, Wm. Crenshaw, Don M. Gallie, G. V. I. Brown, A. H. Peck, J. D. Patterson, B. L. Thorpe.

The Department of Congresses of the Universal Exposition, St. Louis, 1904, has nominated the Committee of Organization of the Fourth International Dental Congress which was appointed by the National Dental Association, and has instructed the committee thus appointed to proceed with the work of organization of said Congress.

Pursuant to the instructions of the Director of Congresses of the Universal Exposition, 1904, the Committee of Organization presents the subjoined outline of the plan of organization of the Dental Congress.

The Congress will be divided into two departments: Department A—Science (divided into four sections). Department B—Applied Science (divided into six sections).

### DEPARTMENT A—SCIENCE.

I. Anatomy, Physiology, Histology, and Microscopy. Chairman, M. H. Cryer, 1420 Chestnut Street, Philadelphia, Pa.

II. Etiology, Pathology, and Bacteriology. Chairman, R. H. Hofheinz, Chamber of Commerce, Rochester, N. Y.

III. Chemistry and Metallurgy. Chairman, J. D. Hodgen, 1005 Sutter Street, San Francisco, Cal.

IV. Oral Hygiene, Prophylaxis, Materia Medica and Therapeutics, and Electro-therapeutics. Chairman, A. H. Peck, 92 State Street, Chicago, Ill.

DEPARTMENT B—APPLIED SCIENCE.

V. Oral Surgery. Chairman, G. V. I. Brown, 445 Milwaukee Avenue, Milwaukee, Wis.

VI. Orthodontia. Chairman, E. H. Angle, 1023 North Grand Avenue, St. Louis, Mo.

VII. Operative Dentistry. Chairman, C. N. Johnson, Marshall Field Building, Chicago, Ill.

VIII. Prosthesis. Chairman, C. R. Turner, Thirty-third and Locust Streets, Philadelphia, Pa.

IX. Education, Nomenclature, Literature, and History. Chairman, Truman W. Brophy, Marshall Field Building, Chicago, Ill.

X. Legislation. Chairman, Wm. Carr, 35 West Forty-sixth Street, New York, N. Y.

COMMITTEES.

Following are the committees appointed:

*Finance.*—Chairman, C. S. Butler, 680 Main Street, Buffalo, N. Y.

*Programme.*—Chairman, A. H. Peck, 92 State Street, Chicago, Ill.

*Exhibits.*—Chairman, D. M. Gallie, 100 State Street, Chicago, Ill.

*Transportation.*—(To be appointed.)

*Reception.*—Chairman, B. Holly Smith, 1007 Madison Avenue, Baltimore, Md.

*Registration.*—Chairman, B. L. Thorpe, 3666 Olive Street, St. Louis, Mo.

*Printing and Publication.*—Chairman, W. E. Boardman, 184 Boylston Street, Boston, Mass.

*Conference with State and Local Dental Societies.*—Chairman, J. A. Libbey, 524 Penn Avenue, Pittsburg, Pa.

*Dental Legislation.*—Chairman, Wm. Carr, 35 West Forty-sixth Street, New York, New York.

*Auditing.*—(Committee of Organization.)

*Invitation.*—Chairman, L. G. Noel, 527½ Church Street, Nashville, Tenn.

*Membership.*—Chairman, J. D. Patterson, Keith and Perry Building, Kansas City, Mo.

*Educational Methods.*—Chairman, T. W. Brophy, Marshall Field Building, Chicago, Ill.

*Oral Surgery.*—Chairman, G. V. I. Brown, 445 Milwaukee Avenue, Milwaukee, Wis.

*Prosthetic Dentistry.*—Chairman, C. R. Turner, Thirty-third and Locust Streets, Philadelphia, Pa.

*Local Committee of Arrangements and Reception.*—Chairman, Wm. Conrad, 3666 Olive Street, St. Louis, Mo.

*Essays.*—Chairman, Wilbur F. Litch, 1500 Locust Street, Philadelphia, Pa.

*History of Dentistry.*—Chairman, Wm. H. Trueman, 900 Spruce Street, Philadelphia, Pa.

*Nomenclature.*—Chairman, A. H. Thompson, 720 Kansas Avenue, Topeka, Kan.

*Promotion of Appointment of Dental Surgeons in the Armies and Navies of the World.*—Chairman, Williams Donnally, 1018 Fourteenth Street N. W., Washington, D. C.

*Care of the Teeth of the Poor.*—Chairman, Thomas Fillebrown, 175 Newbury Street, Boston, Mass.

*Etiology, Pathology, and Bacteriology.*—Chairman, R. H. Hofheinz, Chamber of Commerce, Rochester, N. Y.

*Prize Essays.*—Chairman, James Truman, 4505 Chester Avenue, Philadelphia, Pa.

*Oral Hygiene, Prophylaxis, Materia Medica and Therapeutics, and Electro-therapeutics.*—Chairman, A. H. Peck, 92 State Street, Chicago, Ill.

*Operative Dentistry.*—Chairman, C. N. Johnson, Marshall Field Building, Chicago, Ill.

*Resolutions.*—Chairman, J. Y. Crawford, Jackson Building, Nashville, Tenn.

*Clinics.*—Chairman, J. P. Gray, 212 North Spruce Street, Nashville, Tenn.

*Nominations.*—Chairman, A. H. Peck, 92 State Street, Chicago, Ill.; W. E. Boardman, 184 Boylston Street, Boston, Mass.; M. R. Windhorst, 3518 Morgan Street, St. Louis, Mo.; Wm. Conrad, 3666 Olive Street, St. Louis, Mo.

*Ad Interim.*—Chairman, G. V. I. Brown, 445 Milwaukee Avenue, Milwaukee, Wis.

The officers of the Congress, president, vice-presidents, secretary, and treasurer, will be elected by the Congress at large at the time of the meeting, and will be nominated by the Nominating Committee.

The Fourth International Dental Congress, which will be held August 29 to September 3, inclusive, 1904, will be representative of the existing status of dentistry throughout the world. It is intended further that the Congress shall set forth the history and material progress of dentistry from its crude beginnings through its developmental stages, up to its present condition as a scientific profession.

The International Dental Congress is but one of the large number of congresses to be held during the period of the Louisiana Purchase Exposition, and these in their entirety are intended to exhibit the intellectual progress of the world, as the Exposition will set forth the material progress which has taken place since the Columbian Exposition in 1893.

It is important that each member of the dental profession in America regard this effort to hold an International Dental Congress as a matter in which he has an individual interest, and one which he is under obligation to personally help towards a successful issue. The dental profession of America has not only its own professional record to maintain with a just pride, but, as it is called upon to act the part of host in a gathering of our colleagues from all parts of the world, it has to sustain the reputation of American hospitality as well.

The Committee of Organization appeals earnestly to each member of the profession to do his part in making the Congress a success. Later bulletins will be issued setting forth the personnel of the organization and other particulars, when the details have been more fully arranged.

H. J. Burkhart, *Chairman.*
E. C. Kirk, *Secretary.*

Approved:
Howard J. Rogers,
*Director of Congresses.*
David R. Francis,
*President of Exposition.*

COMMITTEE ON STATE AND LOCAL ORGANIZATIONS.

(J. A. Libbey, Chairman, 524 Penn Avenue, Pittsburg, Pa.)

The Committee on State and Local Organizations is a committee appointed by the Committee of Organization of the Fourth International Congress with the object of promoting the interests of the

Congress in the several States of the Union. Each member of the committee is charged with the duty of receiving applications for membership in the Congress under the rules governing membership as prescribed by the Committee on Membership and approved by the Committee of Organization. These rules provide that *membership in the Congress shall be open to all reputable legally qualified practitioners of dentistry.* Membership in a State or local society is not a necessary qualification for membership in the Congress.

Each State chairman, as named below, is furnished with official application blanks and is authorized to accept the membership fee of ten dollars from all eligible applicants within his State. The State chairman will at once forward the fee and official application with his indorsement to the chairman of the Finance Committee, who will issue the official certificate conferring membership in the Congress. No application from any of the States will be accepted by the chairman of the Finance Committee unless approved by the State chairman, whose indorsement is a certification of eligibility under the membership rules.

A certificate of membership in the Congress will entitle the holder thereof to all the rights and privileges of the Congress, the right of debate, and of voting on all questions which the Congress will be called upon to decide. It will also entitle the member to one copy of the official transactions when published and to participation in all the events for social entertainment which will be officially provided at the time of the Congress.

The attention of all reputable legally qualified practitioners of dentistry is called to the foregoing plan authorized by the Committee of Organization for securing membership in the Congress, and the committee earnestly appeals to each eligible practitioner in the United States who is interested in the success of this great international meeting to make application at once through his State chairman for a membership certificate. By acting promptly in this matter the purpose of the committee to make the Fourth International Dental Congress the largest and most successful meeting of dentists ever held will be realized, and the Congress will thus be placed upon a sound financial basis.

Let every one make it his individual business to help at least to the extent of enrolling himself as a member and the success of the undertaking will be quickly assured. Apply at once to your State chairman. The State chairmen already appointed are:

*General Chairman.*—J. A. Libbey, 524 Penn Avenue, Pittsburg, Pa.

*Alabama.*—H. Clay Hassell, Tuscaloosa.

*Arkansas.*—W. H. Buckley, 510½ Main Street, Little Rock.

*California.*—J. L. Pease, 1016 Clay Street, Oakland.

*Colorado.*—H. A. Fynn, 500 California Building, Denver.

*Connecticut.*—Henry McManus, 80 Pratt Street, Hartford.

*Delaware.*—C. R. Jeffries, New Century Building, Wilmington.

*District of Columbia.*—W. N. Cogan, The Sherman, Washington.

*Florida.*—W. G. Mason, Tampa.

*Georgia.*—H. H. Johnson, Macon.

*Hawaii.*—M. E. Grossman, Box 744, Honolulu.

*Idaho.*—J. B. Burns, Payette.

*Illinois.*—J. E. Hinkins, 131 East Fifty-third Street, Chicago.

*Indiana.*—H. C. Kahlo, 115 East New York Street, Indianapolis.

*Iowa.*—W. R. Clark, Clear Lake.

*Kansas.*—G. A. Esterly, Lawrence.

*Kentucky.*—H. B. Tileston, 314 Equitable Building, Louisville.

*Louisiana.*—Jules J. Sarrazin, 108 Bourbon Street, New Orleans.

*Maine.*—H. A. Kelley, 609 Congress Street, Portland.

*Maryland.*—W. G. Foster, 813 Eutaw Street, Baltimore.

*Massachusetts.*—M. C. Smith, 3 Lee Hall, Lynn.

*Michigan.*—G. S. Shattuck, 539 Fourth Avenue, Detroit.

*Minnesota.*—C. A. Van Duzee, 51 Germania Bank Building, St. Paul.

*Mississippi.*—W. R. Wright, Jackson.

*Missouri.*—J. W. Hull, Altman Building, Kansas City.

*Montana.*—G. E. Longeway, Great Falls.

*Nebraska.*—H. A. Shannon, 1136 "O" Street, Lincoln.

*New Hampshire.*—E. C. Blaisdell, Portsmouth.

*New Jersey.*—Alphonso Irwin, 425 Cooper Street, Camden.

*New York.*—B. C. Nash, 142 West Seventy-eighth Street, New York City.

*North Carolina.*—C. L. Alexander, Charlotte.

*Ohio.*—Henry Barnes, 1415 New England Building, Cleveland.

*Oklahoma.*—T. P. Bringhurst, Shawnee.

*Oregon.*—S. J. Barber, Macleay Building, Portland.

*Pennsylvania.*—H. E. Roberts, 1516 Locust Street, Philadelphia.

*Rhode Island.*—D. F. Keefe, 315 Butler Exchange, Providence.

*South Carolina.*—J. T. Calvert, Spartanburg.

*South Dakota.*—E. S. O'Neil, Canton.

*Tennessee.*—W. P. Sims, Jackson Building, Nashville.

*Texas.*—J. G. Fife, Dallas.

*Utah.*—W. L. Ellerbeck, 21 Hooper Building Salt Lake City.

*Vermont.*—S. D. Hodge, Burlington.

*Virginia.*—F. W. Stiff, 2101 Churchill Avenue, Richmond.

*Washington.*—G. W. Stryker, Everett.

*West Virginia.*—H. H. Harrison, 1141 Main Street, Wheeling.

*Wisconsin.*—A. D. Gropper, 401 East Water Street, Milwaukee.

*For the Committee of Organization,*

EDWARD C. KIRK,

*Secretary.*

MEETING OF THE COMMITTEE OF ORGANIZATION.

At a meeting of the Committee of Organization of the Fourth International Dental Congress held in St. Louis, Mo., April 9, 1904, the following action was taken:

In accordance with the understanding at the last meeting of the committee, held at Washington, D. C., February 23, 1904, that a Nominating Committee for the purpose of nominating officers for the Fourth International Dental Congress be elected at the next meeting of the committee, Dr. M. F. Finley made the following motion:

"That a Committee on Nominations be elected at this time for the purpose of proposing names for the officers of this Congress, and that Drs. A. H. Peck and W. E. Boardman, representing the Committee of the National Dental Association, and Drs. M. R. Windhorst and Wm. Conrad, representing the Committee of the Fédération Dentaire Internationale, be constituted the Committee on Nominations, to present nominations for the officers of the Fourth International Dental Congress, and that said nominations be presented at the present meeting of the Committee of Organization."

The motion was unanimously carried, and Drs. A. H. Peck,

W. E. Boardman, M. R. Windhorst, and William Conrad were elected as the members of the Nominating Committee.

At a subsequent session the Nominating Committee presented the following report:

"Your committee begs to report the following nominations for officers of the Fourth International Dental Congress:

"*President.*—H. J. Burkhart, Batavia, N. Y.

"*Honorary Presidents.*—James Truman, Philadelphia, Pa.; A. H. Fuller, St. Louis, Mo.; G. V. Black, Chicago, Ill.; Thomas Fillebrown, Boston, Mass.; S. G. Perry, New York, N. Y.; Gordon White, Nashville, Tenn.; E. T. Darby, Philadelphia, Pa.; A. W. Harlan, Chicago, Ill.; James McManus, Hartford, Conn.; W. W. Walker, New York, N. Y.; J. N. Crouse, Chicago, Ill.; G. A. Bowman, St. Louis, Mo.; H. A. Smith, Cincinnati, Ohio; T. W. Brophy, Chicago, Ill.; Wm. Jarvie, Brooklyn, N. Y.; Wm. Conrad, St. Louis, Mo.; M. R. Windhorst, St. Louis, Mo.; S. H. Guilford, Philadelphia, Pa.; J. D. Patterson, Kansas City, Mo.; C. C. Chittenden, Madison, Wis.; Wm. Carr, New York, N. Y.; E. H. Smith, Boston, Mass.; M. H. Cryer, Philadelphia, Pa.; E. A. Bogue, New York, N. Y.; V. E. Turner, Raleigh, N. C.; A. L. Northrop, New York, N. Y.; J. H. Moore, Richmond, Va.; C. Newlin Peirce, Philadelphia, Pa.

"*Vice-Presidents.*—A. H. Thompson, Topeka, Kan.; J. G. Reid, Chicago, Ill.; George Fields, Detroit, Mich.; D. O. M. LeCron, St. Louis, Mo.; Garrett Newkirk, Los Angeles, Cal.; R. Ottolengui, New York, N. Y.; R. M. Sanger, East Orange, N. J.; D. N. Rust, Washington, D. C.; N. S. Hoff, Ann Arbor, Mich.; L. P. Bethel, Columbus, Ohio; Jules J. Sarrazin, New Orleans, La.; C. L. Alexander, Charlotte, N. C.; C. H. Darby, St. Joseph, Mo.; B. C. Nash, New York, N. Y.; G. S. Vann, Gadsden, Ala.; B. F. Luckey, Paterson, N. J.; E. R. Warner, Denver, Col.; Williams Donnally, Washington, D. C.; Frank Holland, Atlanta, Ga.; C. A. Meeker, Newark, N. J.; W. P. Dickinson, Minneapolis, Minn.; E. K. Wedelstaedt, St. Paul, Minn.; Adam Flickinger, St. Louis, Mo.; V. H. Jackson, New York, N. Y.; J. M. Whitney, Honolulu, Hawaii; B. Holly Smith, Baltimore, Md.; Louis Ottofy, Manila, P. I.; C. M. Gingrich, Baltimore, Md.; H. B. Tileston, Louisville, Ky.; Wm. Crenshaw, Atlanta, Ga,; J. F. Dowsley, Boston, Mass.; J. W. David, Corsicana, Tex.; Geo. E. Hunt, Indianapolis, Ind.

"*Secretary-General.*—Edward C. Kirk, Philadelphia, Pa.

"*Treasurer.*—M. F. Finley, Washington, D. C.

"*Committee to Nominate Honorary Presidents and Vice-Presidents for Foreign Countries.*—Edward C. Kirk, Philadelphia, Pa.; Edward H. Angle, St. Louis, Mo.; Wilbur F. Litch, Philadelphia, Pa.

(Signed) "A. H. Peck,
"Waldo E. Boardman,
"Wm. Conrad,
"M. R. Windhorst,
"*Committee on Nominations.*"

The above report was adopted by the Committee of Organization subject to ratification by the Congress in general session.

It should be understood that the foregoing list of nominations is necessarily incomplete and subject to future correction and amendment, depending upon individual acceptances and the addition of new names.

Edward C. Kirk,
*Secretary Committee of Organization.*

---

### NEW JERSEY STATE DENTAL SOCIETY.

The thirty-fourth annual session of the New Jersey State Dental Society will convene in the Auditorium, Asbury Park, N. J., at 10 A.M., Wednesday, July 21, 1904, and continue in session Thursday and Friday. Asbury Park is one of the great Atlantic coast watering-places contiguous to New York and Philadelphia. The Auditorium will hold three thousand people, and is open on every side.

Fifty clinics will be given by men from North, South, East, and West most eminent in their profession, and will include the newest advances in all that pertains to operative and mechanical dentistry. In the exhibits the society feels that the latest and largest number of adjuncts to the successful practice of modern dentistry will repay a visit and inspection. The essays will consist of five already accepted and the best obtainable.

The social to members and visiting friends will be, as usual, provided for, and on Thursday evening at 10.30 a smoker will be provided.

The Columbia Hotel will be the head-quarters, with rates of $2.50 to $3.00 per day. Those desiring rooms must send in notices by July 1. The programme, as usual, will be replete with information.

CHARLES A. MEEKER, D.D.S.,
*Secretary.*

29 FULTON STREET, NEWARK, N. J.

---

### FOURTH INTERNATIONAL DENTAL CONGRESS—GENERAL INFORMATION.

THE Fourth International Dental Congress, to be held in St. Louis, August 29 to September 3, inclusive, 1904, will convene in the Coliseum, a building most favorably adapted to the holding of such a gathering and possessing accommodations so ample that all of the features of the Congress will be held under one roof and without interference one with another. This great structure occupies two blocks between Olive and St. Charles, and Thirteenth and Fourteenth Streets; it covers an area of nearly four acres, with a floor space for exposition purposes of three hundred thousand square feet.

The Coliseum is one of the largest and most commodious convention halls ever built, and is practically fireproof. It contains a large theatre capable of seating two thousand five hundred people, which will be used for the general sessions of the Congress, and ten additional meeting-rooms, furnishing ample accommodations for the simultaneous meeting of the ten sectional divisions of the Congress; a large hall for exhibits, covering nine thousand square feet of floor space, practically all of which has been taken by intending exhibitors; and a well-lighted gallery for clinics, capable of accommodating the one hundred chairs which have been provided for that purpose. In addition to the foregoing, numerous committee-rooms and telegraph, telephone, and postal facilities will be provided in the building, and it is expected also that a well-ordered café will be in operation during the time of the Congress.

In connection with the building, and under the same roof, is the Coliseum proper, where nineteen thousand persons may be comfortably seated, exclusive of the stage, and it is anticipated that this audience-room will be used for one of the social features of the Congress, constituting an entertainment unique of its kind.

Besides the advantages of its ample accommodations for the Dental Congress, the Coliseum building has the advantage of being located in the heart of the business section of St. Louis, and at considerable distance from the Exposition, so that the meetings will be less disturbed by the diverting attractions of the great Exposition than if the Congress were held within the Exposition grounds.

ACCOMMODATIONS.

The Local Committee of Arrangements has selected the Hotel Jefferson as the general head-quarters of the Dental Congress. This is one of the most fashionable and complete hotels in the United States, and is located on Twelfth Street, one block from the Coliseum. In addition to the Hotel Jefferson as head-quarters, the hotel accommodations of St. Louis will be sufficient to meet all requirements. The Information Bureau of the Exposition has a list of ninety-seven well-established hotels in St. Louis, with a capacity of forty-one thousand guests, at prices ranging from fifty cents a day upward on the European plan, and from one dollar a day upward on the American plan. These established hotels have been supplemented during the year 1903 by thirty-five new permanent hotels, increasing the permanent hotel capacity to sixty-seven thousand guests, at prices ranging from one dollar a day upward. The Exposition management holds the signed agreement of the leading hotels that "rates shall not be increased during the World's Fair period." Prices are now lower in St. Louis than in any other city for similar hotel accommodations and service.

The Exposition Information Bureau's list of one hundred and thirty-two permanent hotels includes only those of the better class. There are now one hundred and seventy-three hotels, large and small, in operation in the city, and the new hotel enterprises being inaugurated justify the belief that the number will reach two hundred and fifty.

Besides hotels with accommodations for more than two hundred thousand guests, the Exposition Information Bureau has a list of boarding-houses and rooming-houses of a respectable character on the street-car lines with lodgings for sixty-five thousand guests, and a list of private houses that will let rooms for twenty thousand persons. All over the city permanent houses and rooming-houses

THE COLISEUM BUILDING

Where the Fourth International Dental Congress will be held.

are available to those who prefer rooms away from the crowds, with meals at the restaurants. There are four hundred and eighty-five restaurants in St. Louis that have a national reputation for good fare, good service, cleanliness, and moderate prices; twenty of these four hundred and eighty-five restaurants can take care of forty thousand patrons.

### ST. LOUIS AND ITS SURROUNDINGS.

The climate of St. Louis is temperate in summer and most delightful in the spring and autumn. The weather which visitors to the Louisiana Purchase Exposition may expect is shown by the "normals" at St. Louis, taken from the records of the United States Weather Bureau. These "normals" are the averages of the temperatures at St. Louis during the thirty-three years that the Weather Bureau has had a station in St. Louis. The "normals" are as follows: May, 66.1; June, 75.4; July, 79.4; August, 77.6; September, 70.2; October, 58.7; November, 44.3. How closely the actual temperature for any one year follows the normal is well shown by the mean temperature for the month taken by the Weather Bureau at St. Louis during the past year. These temperatures are: May, 71.8; June, 74.2; July, 80.3; August, 76.4; September, 66.4; October, 62.2; November, 63.3. The weather at St. Louis during October and November is particularly pleasant. It is the "Indian summer" of the Middle States.

St. Louis is the most central and most accessible of the four large cities of the United States. Twenty-seven railways enter it, besides passenger steamers on the Mississippi reaching it from the north and south.

World's Fair cheap rates on railways and steamboats will be offered during the whole Exposition season as follows: Season tickets for eighty per cent. of double one fare, good to return until December 15. For sixty days, one and one-third fares, not good to return after December 15. For ten days, one fare plus two dollars, from points within two hundred and fifty miles of St. Louis. For fifteen days, one fare plus two dollars, from points over two hundred and fifty miles from St. Louis.

St. Louis is the fourth city of the United States in point of population, having seven hundred and fifty thousand people. It presents peculiar attractions for the student of nature, science, history, etc. There are twenty-four public parks, containing over two thousand one hundred acres of well-improved property. The World's Fair grounds lie five miles from the Mississippi River on the western border of the State, and are reached quickly and comfortably by steam railways and electric lines. Visitors reach the city through one of the largest railway stations in the world; thirty-two tracks enter the station side by side. Most of the hotels, except those in the World's Fair grounds, are within ten minutes' ride of the station, which is in the heart of the business district. Street-cars reaching all of the hotels for a single five-cent fare pass the station, and the cab, carriage, and baggage system is excellent.

Nearly every member of the Fourth International Dental Congress will wish to see the World's Fair. The Local Committee of Arrangements has already planned a special "Congress day" at the Exposition, and ample opportunity will be provided for members to enjoy visiting this greatest of all expositions. Congressman Bartholdt in a recent speech made before the Congress of the United States, among other things, said, "All in all, the Universal Exposition of 1904 will be the sensational climax of the twentieth century, the grandest victory of peace and civilization, the greatest triumph human genius has yet achieved. To millions of its visitors it will be an academy of learning, an inspiration and an inexhaustible source of genuine delight, and the memories of the 'Ivory City' will live and bear fruit in the ages yet to come."

VISITORS FROM ABROAD.

Extensive preparation is being made for the hospitable care and entertainment of all members attending the Dental Congress. The General Committee of Reception, aided by the local committees, is making every effort to provide for the comfort and care of all visitors. Dr. D. O. M. LeCron, Missouri Trust Building, St. Louis, chairman of the Permanent Local Committee and Bureau of Information, will be pleased to answer all inquiries regarding the accommodations for those who desire to secure them in advance of the Congress. A subcommittee of the General Reception Committee has been appointed to meet and give information and direction to those arriving from Europe and elsewhere at the principal ports of entry of the United States, and to arrange the details of transportation from the sea-board to St. Louis. These committeemen will answer inquiries as to hotels, railways, etc. The subcommittees of the General Reception Committee for the principal ports of entry are:

*New York.*—Drs. W. C. Deane, 114 East Sixtieth Street, and Gladstone Goode, 35 West Forty-sixth Street.

*Philadelphia.*—Drs. J. D. Thomas, 1122 Walnut Street, Joseph Head, 1500 Locust Street, and Julio Endelman, Southeast corner Twelfth and Chestnut Streets.

*San Francisco.*—Drs. H. P. Carlton, 62 Crocker Building, and P. D. Gaskill, Crocker Building.

*New Orleans.*—Drs. J. J. Sarrazin, Godchaux Building, and R. H. Welch, Godchaux Building.

*Baltimore.*—Drs. Cyrus M. Gingrich, 608 St. Paul Street, W. G. Foster, 813 North Eutaw Street, and B. Holly Smith, 1007 Madison Avenue.

In other cities not ports of entry, but which may be visited by members from abroad, the following committeemen will furnish all desired information:

*Buffalo.*—Drs. F. E. Howard, 331 Franklin Street, C. W. Stainton, 47 North Pearl Street, S. Eschelman, 421 Franklin Street.

*Chicago.*—Drs. T. L. Gilmer, 31 Washington Street, J. W. Wassall, 92 State Street, W. V-B. Ames, 31 Washington Street.

*St. Louis.*—Dr. Wm. Conrad, 3666 Olive Street (chairman Local Committee of Reception and Arrangements).

*Washington.*—Drs. H. C. Thompson, 1113 Pennsylvania Avenue, N. W., W. E. Dieffenderfer, 616 Twelfth Street, Williams Donnally, 1118 Fourteenth Street, N. W., and W. N. Cogan, "The Sherman."

The rate for the round trip from New York, exclusive of sleeping-car charge and subsistence, is $32.35. Arrangements have been made for any who may desire to return from St. Louis *via* the Big Four, Lake Shore, and Michigan Southern and the New York Central railways to New York.

### MEMBERSHIP IN THE CONGRESS.

The following are the rules governing membership in the Fourth International Dental Congress, submitted by the Committee on Membership and approved by the Committee of Organization:

1. All reputable practitioners of dental and oral surgery who are entitled to membership in representative State, district, or local dental associations where they reside are eligible for membership in the Congress.

2. The State conference committees in America, and the national chairman of each foreign country have authority to receipt for the membership fee, which, with the application for membership, shall be forwarded to the chairman of the Finance Committee, Dr. C. S. Butler, 680 Main Street, Buffalo, N. Y., who will thereupon forward the official credentials conferring membership in the Congress.

3. If any difference of opinion arises in State committees or national committees as to the eligibility of an applicant for membership, the question shall be referred to the Committee on Membership of the Congress.

4. The wives and children of the members of the Congress may be admitted upon special request and by consent of the Committee on Membership.

5. A uniform fee of ten dollars shall be paid for each membership, and each person whose name appears on the programme either as essayist or clinician must be a paid member of the Congress.

J. D. PATTERSON,
*Chairman Committee on Membership.*

KANSAS CITY, MO., U. S. A.

Membership in the Congress will entitle the holder to all the privileges of debate and discussion of papers, and the right to vote upon all questions which the Congress will be called upon to decide. It will also entitle the members to participate in all the social functions of the Congress under the same conditions as enjoyed by others; to the official badges and insignia of the Congress; to one copy of the complete volumes of the Transactions, which it is anticipated will comprise not less than four volumes of about five hundred pages each. Judging from the material already offered, it is believed that the Transactions of the Congress will be the most complete exposition of modern dentistry yet published. This work will be sent to every member, whether he is able to be present at the Congress or not.

In order to avoid confusion and crowding of work at the last minute, those intending to apply for membership in the Congress are urged to send in their applications at once, which will give time to correct any error should one by chance occur.

All communications of a scientific nature must be submitted to the Committee on Essays for approval before final acceptance for a place upon the programme. All communications to the literary programme of the Congress from foreign countries must receive the approval of the national committee of the respective countries from which they are sent before they can be accepted by the Committee on Essays of the Congress. Each essay must be accompanied by a *résumé* giving the substance of the communication in an epitomized form, which must be in the hands of the Essay Committee thirty days before the opening of the Congress, in order to give opportunity for translation and printing in advance of the Congress, and in order to secure a position upon the official programme. All essays, titles of essays, and *résumés* thereof should be forwarded to Dr. Wilbur F. Litch, 1500 Locust Street, Philadelphia, Pa., U. S. A., or to the secretary of the Committee of Organization.

CLINICS.

All who intend to give clinical demonstrations should communicate with Dr. J. P. Gray, 214 North Spruce Street, Nashville, Tenn., U. S. A., chairman of the Committee on Clinics, who will make the necessary arrangements and supply suitable patients as far as may be possible. The rules governing the approval of literary communications by the several national committees will govern also the clinical demonstrations, and all arrangements for clinical demonstrations must be completed by August 1 in order to secure space and a place upon the programme.

EXHIBITS.

All exhibits of a technical character relating to dentistry will be arranged for by the chairman of the Committee on Exhibits, Dr. D. M. Gallie, 100 State Street, Chicago, Ill., U. S. A., to whom all applications should be made for space. All exhibits relating to dental education will be provided for upon application to Dr. Truman W. Brophy, Marshall Field Building, Chicago, Ill., U. S. A., chairman of Section IX.,—Education, Nomenclature, Literature, and History.

PRIZES.

The Committee of Organization offers two prizes,—viz., a handsome gold medal for the best essay on any subject pertaining to dentistry, and a similar medal for the best exhibit of an archæological character illustrating the development of dental art. All essays in competition for the gold-medal prize are to be forwarded to Dr. James Truman, 4505 Chester Avenue, Philadelphia, Pa., U. S. A., chairman of the Committee on Prize Essays, without the name of the author attached, and designated by a motto, accompanied by a sealed envelope containing the name of the author and bearing upon its outside a duplicate of the motto upon the essay. The committee after having decided upon the respective merits of the essays, and after having selected that one deemed worthy of the medal, will open the envelope bearing the duplicate motto and announce the name of the successful author. The other communications will be destroyed *incognito* six months after the Congress closes unless return of the unsuccessful essays be requested by the authors thereof within that period; or, at the option of the writers, the competing essays which fail to secure the medal may be referred to the Essay Committee for presentation before the Congress. The successful prize essay will be published as a part of the proceedings of the Congress.

The awarding of the prize for the archæological exhibit will be made by a committee to be appointed specially for that purpose. All exhibits competing for this medal will be cared for by the chairman of the Committee on Exhibits, Dr. D. M. Gallie, 100 State Street, Chicago, Ill., U. S. A.

### PRESENT STATE OF ORGANIZATION.

The chairman of the Committee of Organization through Senator Depew has secured from Secretary of State the Hon. John Hay a promise to send through our foreign ambassadors and representatives an invitation, on behalf of our government, to all governments with which the United States is in diplomatic relation to send an official delegate to the Congress, and the Secretary has received notification that these invitations have been issued.

Upward of twenty nations have signified their intention to take part in this great Congress. No fewer than fifteen hundred committeemen are now actively at work promoting the success of the meeting. Every State and Territory in the United States is in charge of a State committee actively at work in developing the details of the Congress in a local way. So that the prospect of an unusually large attendance is practically assured, and it is confidently expected that the membership in the Fourth International Dental Congress will be much in excess of any other dental meeting ever held. The number and character of essays already prepared, the number and character of the clinical demonstrations, the magnitude of the exhibits already arranged for, will surpass in these features all previous dental meetings. The work which has been accomplished by the Committee on Education, Legislation, and Dental History will constitute the most extensive contributions to these departments yet made.

The social features of the Congress are being provided for upon an elaborate plan. Receptions, lunches, and various forms of entertainment are being arranged on a scale commensurate with the magnitude and importance of the meeting, and as much time will be given to the amenities of social intercourse as may be consistent with the more serious features of the programme.

The Fourth International Dental Congress is now an assured success, and, judged from any stand-point, it will be a meeting which will not only adequately set forth the most recent developments of dental science and art, but it will constitute a liberal education in dentistry which no progressive practitioner can afford to miss.

An efficient corps of interpreters has been provided to assist those visiting members who are unfamiliar with the English language.

EDWARD C. KIRK,
*Secretary Committee of Organization.*

---

### DENTAL COMMISSIONERS OF CONNECTICUT.

THE Dental Commissioners of the State of Connecticut hereby give notice that they will meet at Hartford, on Thursday, Friday, and Saturday, July 14, 15, 16, 1904, respectively, to examine applicants for license to practise dentistry, and for the transaction of any other proper business.

The practical examination in operative and prosthetic dentistry will be held Thursday, July 14, at 9 A.M., in Putnam Phalanx Armory, corner Haynes and Pearl Streets.

The written theoretic examination will be held Friday and Saturday, July 15 and 16, at the Capitol.

All applicants should apply to the Recorder for proper blanks, and for the revised rules for conducting the examinations.

Application blanks must be carefully filled in and sworn to, and with fee, twenty-five dollars, filed with the Recorder on or before July 7, 1904. Examination Fee must be forwarded by Money Order or Certified Check.

By direction of the Dental Commissioners.

J. TENNY BARKER,
*Recorder.*

### COLORADO STATE BOARD OF DENTAL EXAMINERS.

The regular semi-annual meeting of the Colorado State Board of Dental Examiners will be held in Denver, June 7, 8, and 9, 1904. The examination will be both theoretical and practical, and applicants for the examination must be prepared to do such practical work as is required. All applications must be filed before June 7. For particulars address,

M. S. Fraser, *Secretary,*
407 Mack Building, Denver, Col.

---

### GEORGIA STATE DENTAL SOCIETY.

The thirty-sixth annual meeting of the Georgia State Dental Society will be held in Athens, Ga., June 28, 29, and 30, 1904. Arrangements are being made to make this the greatest convention ever held in Georgia. All ethical practitioners are cordially invited.

A. M. Jackson,
*President.*
D. H. McNeill,
*Corresponding Secretary.*

---

### NORTHERN OHIO DENTAL ASSOCIATION.

The forty-fifth annual meeting of the Northern Ohio Dental Association will be held in Cleveland, Tuesday, Wednesday, and Thursday, June 7, 8, and 9, 1904. The programme is a strong one, and will be of exceptional interest to the general profession. The motto for the year is "Annihilation of Pain in Dentistry." Essayists and clinicians have been selected with this thought ever foremost. The best authorities and the most successful men in this line of work will be at this meeting. The members of the profession are cordially invited to attend. It is expected that we will have the largest attendance of any meeting ever held in this section of the country. You cannot afford to miss it. Come!

W. G. Ebersole,
*Corresponding Secretary.*

## Figures and Tables

**Figure f1:**